# Cognitive Loading Affects Motor Awareness and Movement Kinematics but Not Locomotor Trajectories during Goal-Directed Walking in a Virtual Reality Environment

**DOI:** 10.1371/journal.pone.0085560

**Published:** 2014-01-21

**Authors:** Oliver Alan Kannape, Arnaud Barré, Kamiar Aminian, Olaf Blanke

**Affiliations:** 1 Laboratory of Cognitive Neuroscience, Brain Mind Institute, School of Life Sciences, École Polytechnique Fédérale de Lausanne (EPFL), Lausanne, Switzerland; 2 Laboratory of Movement Analysis and Measurement, Institute of Bioengineering, École Polytechnique Fédérale de Lausanne (EPFL), Lausanne, Switzerland; 3 Center for Neuroprosthetics, School of Life Sciences, École Polytechnique Fédérale de Lausanne (EPFL), Lausanne, Switzerland; 4 Department of Neurology, University Hospital, Geneva, Switzerland; University of Tokyo, Japan

## Abstract

The primary purpose of this study was to investigate the effects of cognitive loading on movement kinematics and trajectory formation during goal-directed walking in a virtual reality (VR) environment. The secondary objective was to measure how participants corrected their trajectories for perturbed feedback and how participants' awareness of such perturbations changed under cognitive loading. We asked 14 healthy young adults to walk towards four different target locations in a VR environment while their movements were tracked and played back in real-time on a large projection screen. In 75% of all trials we introduced angular deviations of ±5° to ±30° between the veridical walking trajectory and the visual feedback. Participants performed a second experimental block under cognitive load (serial-7 subtraction, counter-balanced across participants). We measured walking kinematics (joint-angles, velocity profiles) and motor performance (end-point-compensation, trajectory-deviations). Motor awareness was determined by asking participants to rate the veracity of the feedback after every trial. In-line with previous findings in natural settings, participants displayed stereotypical walking trajectories in a VR environment. Our results extend these findings as they demonstrate that taxing cognitive resources did not affect trajectory formation and deviations although it interfered with the participants' movement kinematics, in particular walking velocity. Additionally, we report that motor awareness was selectively impaired by the secondary task in trials with high perceptual uncertainty. Compared with data on eye and arm movements our findings lend support to the hypothesis that the central nervous system (CNS) uses common mechanisms to govern goal-directed movements, including locomotion. We discuss our results with respect to the use of VR methods in gait control and rehabilitation.

## Introduction

Dual tasking (DT) paradigms have provided compelling evidence in favour of cortical involvement in the sensorimotor control of balance and locomotion in humans [1], [2]. Cognitive tasks such as verbal fluency [3], fine-motor movements (e.g. buttoning up [4]) and arithmetic [5] have been shown to alter gait characteristics ranging from walking velocity, over stride-variability to stride-asymmetry during over-ground and treadmill walking. Such gait changes during dual tasking are more pronounced in elderly with fall risk and are used as a marker for age-related decline in gait control [6], [7]. While the effects of cognitive loading on movement kinematics are well documented, little is known about its influence on goal-directed walking behaviour. Notably, the vast majority of everyday tasks, such as picking up the morning paper, involve a series of goal-directed movements. We visually scan the room for the paper; we reach for the paper and, if it is on the other side of the room, we walk towards the paper, avoiding the sleeping dog. Striking similarities have been reported between the trajectories made for saccadic eye and arm movements [8] but also between arm movements in the sagittal plane and vertical whole-body movements [9]. Pham and Hicheur [10], [11] reported stereotypical trajectories during goal-directed walking similar to those reported for upper-limb reaching movements. Based on these data it has been suggested that the central nervous system (CNS) may employ a common strategy to govern goal-directed behaviour, for example by minimising the variance in the final position [8]. For goal-directed walking any such strategy appears to be linked to the formation of whole-body trajectories rather than the co-ordination of a sequence of steps [12] and it is currently not known how this (strategy) is affected by taxing cognitive resources.

We have previously reported participants' walking performance but also awareness of their motor performance in a goal-directed walking paradigm in a virtual reality (VR) environment [13]. These results showed that participants compensated for introduced visual angular deviations of up to 15° without becoming aware of either the sensorimotor mismatch or their corrective movements. With deviations upwards of 15° participants reported a switch as they consciously compensated for the introduced angular deviations. The goal of the current study was to extend this paradigm to investigate the role of cognition (using the serial 7 subtraction task) in the execution of goal-directed locomotion, as illustrated by the participants' walking trajectories, the motor implementation, as measured through the movement kinematics, as well as its influence on motor awareness. We investigated this by employing techniques from VR including full-body motion capture and real-time visual movement feedback.

## Methods

### Participants

Fourteen healthy, adult participants volunteered for the study (7 male, 7 female, mean age = 23±6 years, height = 173±10 cm, weight = 64±13 kg). Participants had normal or corrected to normal vision.

### Ethics Statement

The study was conducted according to the principles expressed in the Declaration of Helsinki and approved by the local ethics committee – La commission d'éthiqe de la recherche Clinique de la Faculté de Biologie et de Médecine at the University of Lausanne, Switzerland. All participants provided written informed consent for the collection of data and subsequent analysis. Anonymized data is available upon individual request and in accordance with the local ethical committee's guidelines.

### Materials

**Figure 1:**
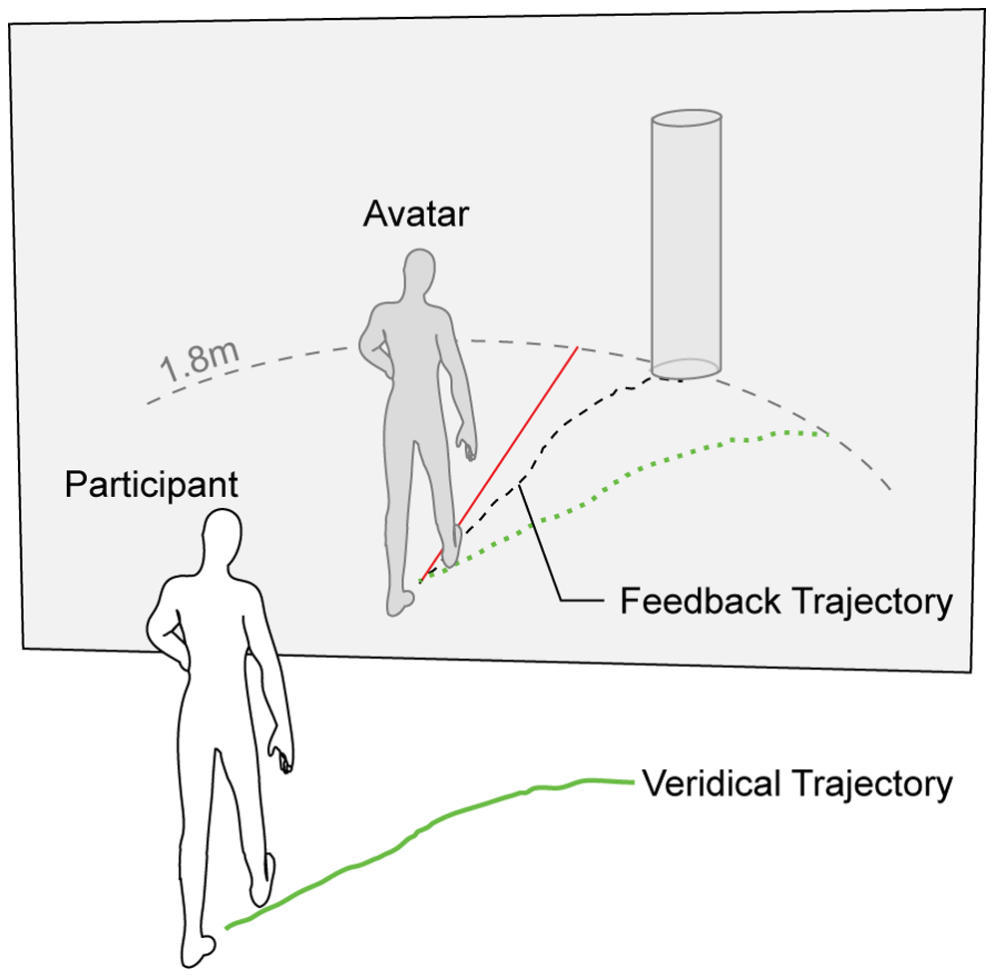
Experimental Setup. Participant movements were recorded using an optical motion capture system, mapped to a life-size avatar and played back in real-time on a rear-projection screen. In each trial a participant walked from a fixed start position to one of four randomized target positions set along a 1.8 m perimeter. In some trials an angular deviation of ±5° to ±30° (red line) was introduced between the participant's veridical walking trajectory (green line, solid and dotted) and the feedback trajectory (dashed black line). At the end of each trial participants further judged whether the movement feedback they received corresponded to their actual movement. Participants performed one block each with or without cognitive loading.

**Figure 2:**
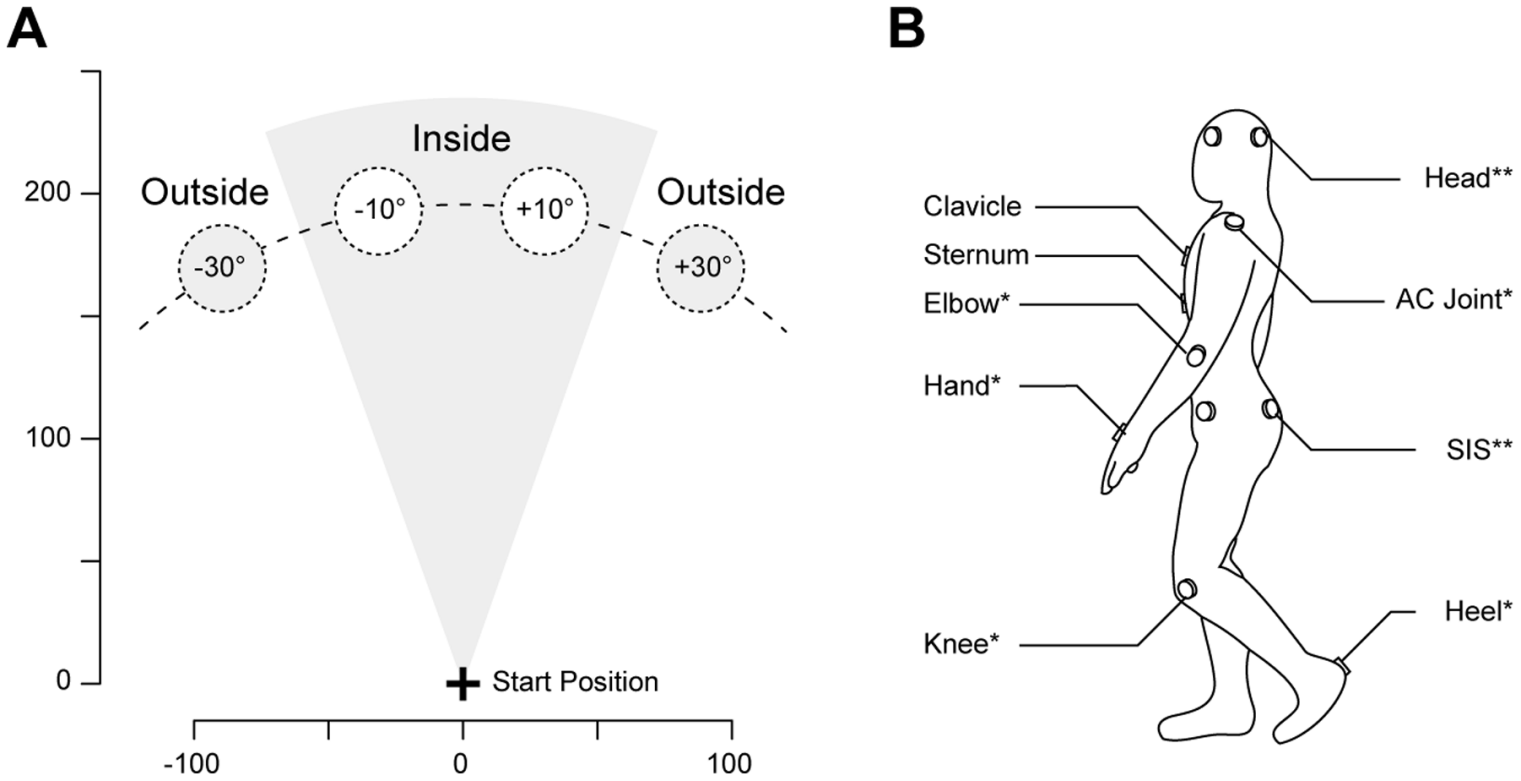
Target Positions and Marker Placement. **A** Motion capture area as seen from above. The four targets are placed at a distance of 180±10° (inside) and ±30° (outside). The start position was indicated in the real room but the final position of the target was recalculated using the exact location at button-press. **B** Participants wore 20 infrared markers: One each on the sternoclavicular joint and the lower sternum, *2 on left-right heel, lateral knee and elbow, dorsal hand and acromioclavicular (AC) joint, **4 on left-right, anterior-posterior superior iliac spine (SIS) and head. Walking trajectories were determined by the average SIS marker position.

Participants' movements were tracked and recorded by an active optical motion capture system (20 IR markers, ReActor2, Ascension Technology Corp., Burlington, VT, USA) at a sampling frequency of 30 Hz. A schematic of the setup and task is illustrated in Figure 1. Target positions and marker placements are indicated in Figure 2 A and B. Participants received visual feedback of their movements by way of a 3.20 m×2.35 m back-projection screen (width×height, 1280×1024 pixels, 60 Hz), with the screen itself forming part of the back-wall of the 4.11×4.11 m tracking arena (projector: JVC DLA-SX21 projector, JVC U.S.A., Wayne, NJ, USA). In each of the 176 trials (2 blocks of 88trials), participants viewed an individually mapped, life-size virtual body perform their movements in real-time (intrinsic delay 75 ms).

### Experimental Procedure

Participants performed two experimental blocks, a single task session (ST) and a dual task session (DT), counterbalanced across participants. The experimental procedure is illustrated in Figure S1. Each trial started from a predefined location in the motion capture area. A semi-transparent target cylinder was shown in the virtual room at one of four randomised locations (see Figure 2A) as shown on a rear-projection screen. Participants were asked to walk through the virtual target with their virtual body by walking in the motion capture area. In some trials, in randomized order and beyond a distance of 30 cm from the start location, the walking trajectory of the virtual body was systematically deviated towards either the left or the right (by 5°, 10°, 15° or 30°) [13]. The deviation of the virtual trajectory was calculated relative to the straight line between the participants' current position and the position of deviation onset. Direction and amplitude were randomized on a trial-by-trial basis. A trial ended as soon as the participant reached the target distance of 180 cm, independent of reaching the centre of the target cylinder. Subsequently, participants indicated using a joystick whether the feedback shown on the screen corresponded to the movement they had just performed [14]. In the dual task experimental block participants performed the same walking task while performing an articulated arithmetic task (serial-7 subtractions, counterbalanced design, 88 trials per block, including 24 control trials, i.e. no deviation, and 16 trials per deviation, randomized but evenly distributed across direction and targets). Participants were instructed to continuously count and only stop while responding to the agency attribution question. They started counting from 200 and continued counting backwards throughout the entire block, ensuring that the cognitive load commenced before and lasted throughout each trial. We chose the serial-7 subtraction task, as it has been reported to cause gait changes such as a decrease in velocity in young healthy participants as well as patient populations [15], an increase in stride-length and stride-time in healthy elderly [5] and patients and increased gait variability in neurological patients with Parkinson's disease [16], [17].

### Gait Analysis

The biomechanical model used for gait analysis is derived from the Plugin Gait Markers set. The hip joint centres were determined by using regressions equation [18]. The pose of the segmental frames for the head, the trunk and the pelvis during the dynamic acquisition were determined by a point clouds fitting method [19] using as reference the registered position of the markers affixed on each segment and measured during a static calibration. The 3D joint angles for the neck (head relative to the trunk) as well as for the thorax (trunk relative to the pelvis) were decomposed using the Cardan sequence ZX′Y″. The global angles for the head, trunk and the pelvis were determined by the global sequence YXZ. The angles for the knees (shank relative to the thigh) were determined in the plane built by the two segments used. The same method was used for the angle of the elbows (forearm relative to the upper arm) yielding the flexion-extension angles for these joints. For each subject, an additional baseline correction was performed.

#### Motor Performance

We described the total angle compensated by the participant taking into account the endpoint of each of their movement trajectories and measured from the onset of deviation at a distance of 30 cm to the start location as indicated in Figure S2A.

*Motor Performance [°]:*

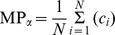



*c = compensation; α = angular deviation; N = number of trials*

The mean position of the four hip markers was used to analyse all walking trajectories. Trials that were longer in duration than 10 seconds and trials that were corrupted through marker occlusions were omitted. All trajectories were interpolated over both time and space to 300 samples each. Furthermore the trajectories were rotated from their four target locations at β = (−30°, −10°, 10°, 30°) and overlapped onto a single target by transforming their samples into polar coordinates, rotating them, and returning them into the Cartesian coordinate format.

*Cartesian to Polar:*

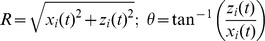



*x = x-coordinate; z = z-coordinate; i = trial index; t = sample;*

*R = radial coordinate; θ = angular coordinate*

*Polar to Cartesian:*

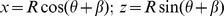



*β = target location*

#### Mean Walking Trajectory

The mean trajectory was obtained by taking the arithmetic mean of the x and z coordinates at each sample across all trials with the same angular deviation:

*Mean Trajectory:*

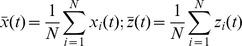



*N = number of trials*

The average trajectory deviation (ATD), Figure S2B, was determined by averaging across the distance between each coordinate-pair of the single and the mean trajectory for the same angular deviation. As the ATD is calculated sample by sample it takes participants' timing into account. In other words the trajectory deviation increases both for differences in the x-z plane as well as in the timing or velocity of each walking trajectory. The maximum trajectory deviation (MTD) was obtained by keeping only the value of the maximal deviation for each trial. We additionally defined an ideal trajectory for each angular deviation in order to have an objective and time-independent measure as explained in detail below. Samples that were two standard deviations above or below the average were removed from the calculation of mean and maximum deviation.

#### Coefficient of Variance

The coefficient of Variance (CV) was defined as the ratio of the standard deviation (σ) of a given variable to its mean (μ):
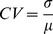



#### Ideal Trajectory

Furthermore, an ideal trajectory was defined for each angular deviation (α) as an overall, objective reference, independent of walking speed. Each ideal trajectory was composed by a set of two linear functions; first, the straight line towards the target, second, a line that took into account the angular deviation introduced (Figure S2c). The ideal trajectory had to exactly compensate for the deviation, which was introduced at a distance of 30 cm from the trial's starting point. An error signal was then obtained to determine average and maximum trajectory deviations. The point on the ideal trajectory whose orthogonal crosses the sample was used to determine the shortest distance between ideal and actual trajectories, which in turn was used as the error signal.

*Ideal Trajectory:*

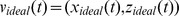


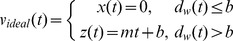



*m = tan(90+α);b = 30; d_w_(t) = Walking Distance (see below)*

*Sample Distance:*

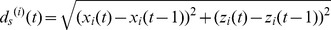



*Walking Distance:*

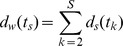



*S = Number of samples (300)*

The ideal trajectories were used to calculate the average trajectory error for the individual angular deviations.

*Deviation (Sample Error):*

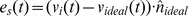



*n_ideal_ = unit normal vector of v_ideal_*

*Average Trajectory Error:*

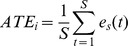



#### Time to Target and Velocity

The time and exact location of the participant at the press of the start button is used as the trial coordinate origin and start-time. Time to target is therefore the difference between the time-stamp of the first motion-capture sample that is further than 180 cm away from the start position and the trial start-time. The distance of the x-z location from this sample to the origin describes the exact distance the participant walked. The average walking velocity is determined by their ratio: distance over time in meters per second. Similarly, the velocities and durations for the start, middle, and end of the trial are calculated using the motion-capture coordinates and time-stamps as participants cross 30, 150, and 180 cms.

### Motor Awareness Analysis

Motor Awareness (MA) was expressed by the number of yes-responses out of all valid trials, grouped by angular deviation [13]. Correct MA or self-attribution was a “yes” response for non-deviated, a “no” response for a deviated trial. Additionally, MA thresholds were determined psychometrically, by fitting a cumulative Gaussian to the participants' responses using the published psignifit toolbox [20], [21] for Matlab. All thresholds reported here reflect the 50% point of subjective equality.

*Motor Awareness [%]:*

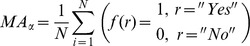



*r = response*

### Statistical Analysis

Motor awareness and gait characteristics were recorded throughout the entire study and processed offline using R [22] and Matlab (MathWorks, Natick, Massachusetts, USA).

To investigate the overall effects of Task and Deviation in-line with our previous studies, we first collapsed all trials into a single target location and included both control trials and (absolute) deviated trials. This resulted in a 2×5 repeated measures ANOVA with factors *Task* and *Deviation* and levels ST, DT and 0°, 5°, 10°, 15°, 30° respectively. In a second step, we separated the 0° control trials from the (signed) deviated trials in order to investigate possible laterality effects of deviation and target, target positions, and possible interactions. This resulted in a 2×2×2×2 rmANOVA with factors *Task* (ST/DT), *Target Side* (l/r), *Target Position* (in/out), and *Deviation Side* (l/r). As factor Task was included in the first set of ANOVAs these results were not included. RM ANOVAs and post-hoc comparisons, Fisher's LSD, were performed in Statistica (StatSoft, Tulsa, Oklahoma). The psychometric data were compared using Student's t-test. One participants' neck yaw data was omitted from analysis due to a corrupt head marker.

## Results

Of 88 trials per condition, 84.75 were included in the analysis (on average per person and condition). Slightly, but significantly more trials were rejected in the DT condition 84.0+/−3.0 (DT) versus 85.5+/−1.7 (ST, paired t-test p = 0.041). A summary of the main results is provided in table 1.

**Table 1:** Results Overview.

		Angular Deviation					Condition			Statistics	
		0°	5°	10°	15°	30°	ST	DT	Task	Deviation	Task by Deviation
**Walking Trajectories** *(mean±SEM)*	MP [°]	1.04±.25	3.33±.14	5.18±.33	7.66±.54	14.47±1.23	7.60±.55✠	7.72±.56✠	p>.64	p<.001***	p>.79
	ATD [cm]	8.62±.59	9.65±.71	10.16±1.00	9.64±.61	11.48±.86	9.27±.56	10.55±.95	*p>.06*	p<.001***	p>.54
	MTD [cm]	45.15±3.80	46.01±4.33	48.80±4.24	46.51±4.96	52.05±3.89	43.28±3.19	52.13±4.56	p = .032*	p>.41	p>.14
	ATE [cm]	6.33±.51	6.85±.52	8.31±.43	9.95±.46	19.42±.72	10.25±.42	10.09±.50	p>.54	p<.001***	**p = .039**
**Kinematics**	WT [sec]	3.61±.10	4.13±.43	4.14±.33	4.19±.19	4.38±.67	3.84±.89	4.34±.92	p = .016*	p<.001***	p = .124
	Neck [rel. °]	7.53±.52	7.60±.59	7.70±.60	7.79±.54	8.60±.78	7.84±.64	7.85±.72	p>.98	p<.001***	*p>.08*
	Neck CV	0.79±.05	0.77±.05	0.80±.05	0.79±.04	0.83±.05	0.80±.04	0.79±.06	p>.69	p>.19	p>.31
	Knee [rel. °]	7.30±.31	7.00±.27	7.20±.27	7.34±.37	7.11±.29	7.32±.27	7.06±.34	p>.21	p>.09	*p>.05*
	Knee CV	1.20±.06	1.16±.05	1.18±.06	1.21±.06	1.20±.06	1.22±.06	1.16±.06	p>.31	p>.63	p>.70
**Motor Awareness**	MA✠✠ [%yes]	95.8±1.1	92.3±1.9	73.5±4.9	46.5±6.5	7.4±2.4	61.2±2.8	65.1±2.9	*p>.06*	p<.001***	p = .01*
	RT [sec]	1.36±.13	1.51±.15	1.58±.17	1.58±1.46	1.46±.13	1.48±.14	1.52±.15	p>.64	p>.25	p>.16

Walking trajectories were not significantly impacted by the cognitive load and clearly dependent on the introduced angular deviations in both experimental conditions. The main effect of Task on maximum trajectory deviations is due to the fact that the timing of the trials is integrated in these calculations. Walking time (WT), i.e. velocity was significantly affected in the DT condition as participants systematically slowed down. Motor awareness strongly depended on the angular deviations and, in trials around the perceptual threshold, significantly declined in the DT condition.

mean over deviated trials only;

Motor Awareness significantly affected by the Dual Task for angular deviations of 10° and 15°.

(* p<.05, ** p<.01, *** p<.001).

### Motor Performance

#### Motor Compensation at Trajectory Endpoint

**Figure 3:**
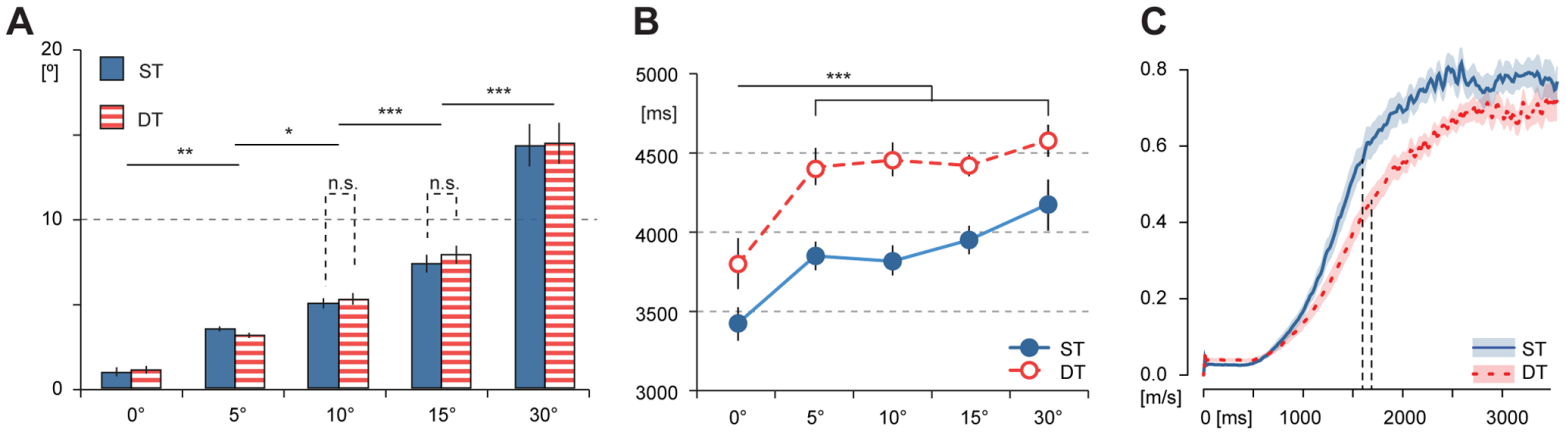
Motor Performance Overview. **A** Motor Compensation– Participants consistently compensated for the introduced angular deviation as MP monotonously increased with the deviation. The secondary task had no effect on this compensation, even in trials corresponding to the highest perceptual uncertainty (10° and 15°). **B** **Time to Target** – Participants were significantly slower in the dual task condition than in the single task condition. Independent of cognitive loading participants were significantly faster in the 0° control trials. **C** **Average Velocity Profile.** The velocity profile shown here is averaged across all trials and participants. Participants slowed down significantly as a result of the secondary cognitive task, articulated serial-7 subtraction. We did not observe an initial freezing-like behaviour as there was no change in the time participants took to cross the first 30 cm of each trial, as indicated by the dotted black lines. Instead, walking velocity was lower over the entire trial. Error bars are standard error of the mean (SEM).

Overall, as illustrated in Figure 3A, participants correctly performed the walking task as motor compensation increased proportionally to the introduced angular deviation (main effect of Deviation: F(4, 52) = 110.10, p<0.001, cf. Table S1 for post-hoc comparisons). Cognitive loading had no significant effect on this motor compensation (average compensation ST: 7.6°±.5, DT: 7.7°±.5, main effect of Task p>0.64; interaction: p>0.79). On average participants compensated for 54±2% of the introduced deviation.

In the non-deviated (0°) control trials, participants accurately walked to within 1.0°±0.3° of the centre of the target in the ST and 1.1°±0.2° in the DT condition. Motor performance was very stable in control trials and was not influenced by the independent factors Target Side and Target Position (p>.39 and p>.18 respectively, all interactions p>.26).

As observed in the control trials, Motor Performance in deviated trials was not affected by Task or Target Side (p>0.57 and p>0.75 respectively). However, motor performance did depend on Target Position (F(1, 13) = 6.5548, p = .02373). Participants compensated more accurately when walking towards the inside targets (7.6±0.5°) than when walking towards the outside targets (7.0±0.5°). Moreover, we observed a three-way interaction between factors Target Side×Target Position×Deviation Side (F(1, 13) = 11.035, p = .00551). Participants were more accurate when walking towards the outside targets, if the compensation was towards the inside or midline, i.e. when walking towards the leftmost (rightmost) target with a deviation to the left (right). This relationship was flipped for the inside targets. Here participants were more accurate, if the deviation was towards the centre of the tracking arena and they compensated outwards.

We further observed an interaction between factors Task and Target Side (F(1, 13) = 5.3147, p = .03828); MP was increased for the targets on the left-hand side when walking under cognitive load but decreased for targets on the right-hand side (all post-hoc comparisons: p>0.12). Finally, there was an interaction between factors Task, Target Position, and Deviation Side (F(1, 13) = 5.2380, p = .03948).

#### Time to Target and Velocity Profiles

The average walking time to reach the target significantly increased from 3.8±0.09 seconds in the ST condition to 4.3±0.09 seconds in the DT condition when considering all trials (ν_ST_ = 0.46±0.07 m/s and ν_DT_ = 0.40±0.08 m/s from a standing start, see Figure 3B and C). This was confirmed in the rmANOVA that yielded main effects of Task (F(1, 13) = 7.5503, p = 0.016) and Deviation F(4, 52) = 18.212, p<0.001). The latter effect resulted from significantly faster walking times in the 0° control trials as well as significantly slower times for ±30° trials, see Table S2. Target Position (p>0.92) and Target Side (p>0.55) did not affect walking times. There was no significant interaction between factors Task and Deviation (p = 0.124).

We further analysed the deviated trials separately in order to check for laterality effects. While there was no main effect of Deviation Side (p>0.08), we observed an interaction between Target Side and Deviation Side (F(1, 13) = 5.7145, p = .03266). Participants took longer to complete trials to targets on the left hand side when the deviation was also towards the left hand side and vice versa.

We additionally analysed the first 30 cm of the walking trajectories to exclude an initial hesitation or “freezing” as cause for changes in MA. As illustrated in Figure 3C, there was no significant difference in mean velocity for this segment between the two conditions and hence no significant difference in time to reach 30 cm (ST: 1614±70 ms, DT: 1684±62 ms, paired t-test: p>0.2). Participants were significantly faster in the ST condition than in the DT condition for the middle segment (31–150 cm, p<0.001) and end segment (151–180 cm, p<0.001).

#### Average Trajectories, Deviations and Errors

**Figure 4:**
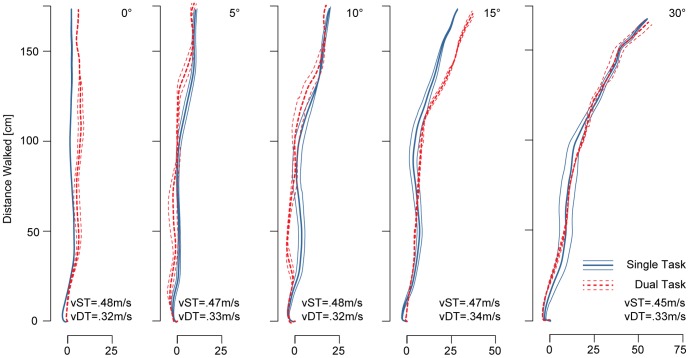
Walking Trajectories. Mean walking trajectories are illustrated for a single subject, averaged for each angular deviation; dotted lines indicate standard deviations. Participants' compensation for introduced angular deviations resulted in stereotypical walking trajectories. Importantly, these trajectories were not significantly affected by the introduction of a cognitive load even though the average walking velocity significantly decreased.

Figure 4 shows the average trajectories of a single participant for the five angular deviations. The average trajectory deviation (ATD) across all participants was 9.3±0.6 cm in the ST condition and slightly increased to 10.6±0.9 cm under cognitive loading (main effect of Task: F(1, 13) = 4.1358, p = .06292 not significant). There was a main effect of Deviation (F(4, 52) = 9.4949, p = .00001) as ATD was lowest for control trials (8.6±0.6 cm) and highest for trials with ±30° deviations (11.5±0.9 cm). There was no interaction between factors Task and Deviation (p>0.55). Maximum trajectory deviation (MTD) increased from 43.3±3.2 cm in the ST condition to 52.1±4.6 cm in the DT condition (main effect of Task: F(1, 13) = 5.8044, p = .03154). MTD was again lowest for control trials (ST: 36.9±3.5, DT: 53.4±6.1 cm). There was no main effect of Deviation on MTD (p>0.41, cf. table 1).

Our results from the average trajectory error (ATE) illustrate that walking trajectories, measured with respect to an ideally compensatory trajectory, were not significantly affected by cognitive loading (p>0.54). However, the ATE significantly depended on the angular deviation, reflecting the constant gain observed for the motor compensation (main effect of Deviation: F(4, 52) = 254.30, p = 0.0000). The average trajectory error thus increased from 6.3±0.5 cm in control trials to 19.4±0.7 cm in trials with 30° deviation. We further observed an interaction between factors Task and Deviation (F(4, 52) = 2.7346, p = .03856); ATE was slightly higher in the DT condition for deviations of 0°, 5°, and 10°, but lower for deviations of 15° and 30°.

#### Neck Yaw

In the current paradigm we were especially interested in the relative axial rotation angles between the head and torso (neck yaw), which describes the heading direction. Overall, neck yaw was strongly affected by the magnitude of the deviation as participants turned their heads more pronouncedly with increasing deviation (main effect of Deviation: F(4, 48) = 6.3773, p = .00034). Neck yaw thus monotonously increased from 7.53°±0.52 to 8.60°±0.80 and post-hoc comparisons illustrated that neck yaw was significantly higher for the ±30° deviations (all comparisons to ±30° p<0.016, all others p = 1, Bonferroni corrected, cf. Table S3). The secondary task had no significant effect on neck axial rotations (main effect of Task: p = 0.98) and there was no significant interaction between factors Task and Deviation (p>0.089).

Neck yaw in control trials did not depend on the side of the target (main effect of Target Side: p>0.4). In-line with the absolute position of the virtual target with respect to the feedback screen, we observed a significant effect of target position (main effect of Target Position: F(1, 12) = 50.816, p = .00001). Participants turned their head more when walking towards the outside targets (yaw: 9.4°±0.7) than the inside targets (yaw: 5.6°±0.3).

Similarly, neck yaw in deviated trials illustrated a strong main effect of Target Position (F(1, 12) = 52.524, p = .00001) as yaw was significantly higher when walking towards the outside targets and turning towards the midline than when walking towards the inside targets. This was similar for left and right targets (main effect of Target Side: p>0.52 n.s.). We further observed a strong interaction between factors Target Side and Deviation Side (Current effect: F(1, 12) = 21.723, p = .00055): participants turned their head less when leftwards deviations coincided with targets on the left (so they compensated towards the midline) and vice versa.

We observed small but significant interactions between factors Target Position and Deviation Side (F(1, 12) = 5.0137, p = .04487) as well as factors Task, Target Position and Deviation Side (F(1, 12) = 6.8266, p = .02268). None of the other interactions were significant (all p>0.05).

#### Summary Motor Performance

In summary, participants were able to accurately perform the goal-directed walking task. Motor compensation, as measured at the trajectory endpoint, was very accurate for 0° control trials and unaffected by the Task, Target Side or Target Position. Accordingly, motor compensation increased relative to the introduced deviation. In deviated trials, MP was more accurate for inside than for outside targets but still unaffected by cognitive loading. In-line with the dual tasking literature, cognitive loading had a main effect on the walking velocity as participants significantly slowed down in the dual task condition. Importantly, this strong influence of taxing cognitive resources was not reflected in motor compensation or the walking trajectories as described by the trajectory deviations and the trajectory error. The trajectory deviations recorded in a virtual environment were comparable to those previously reported in a natural environment. Trajectory deviations significantly increased with the introduced deviation. Furthermore, neck yaw, indicating heading, was susceptible to the position of the target and the side of the target in combination with the side of the deviation, but again unaffected by cognitive loading.

### Motor Awareness

**Figure 5:**
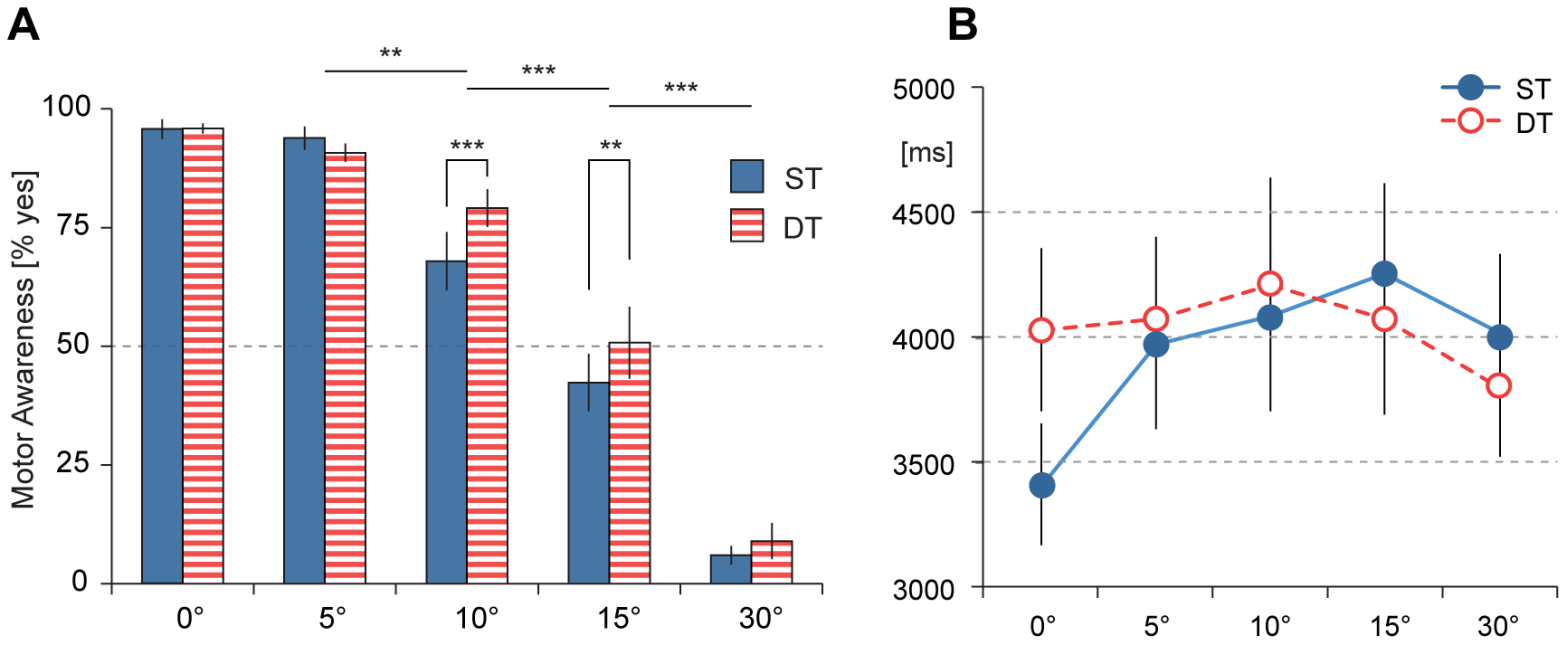
Motor Awareness and Response Times. **A** Participants correctly judged feedback in 0° control trials to be true. This self-attribution significantly decreased with increasing angular deviations. In case of the largest deviations of ±30° participants correctly rejected almost all trials as deviated. Cognitive loading significantly impaired motor awareness for trials with deviations of ±10° to ±15° as participants judged significantly more trials to be non-deviated than in the single task condition. **B** Response times (RT) were recorded for all trials. Participants were instructed to respond promptly but the priority was placed on completing the task correctly. In the single task condition participants replied fastest for the 0° control trials, with RTs increasing almost linearly with increasing deviation. RTs decreased for trials with the largest deviations of 30° in both the ST and DT condition. All error bars are SEM.

As illustrated in Figure 5A participants correctly judged 95.7±2.0% (mean ± SEM) of non-deviated trials to be self-generated. This percentage monotonously dropped with increasing angular deviations (main effect of Deviation: F(4, 52) = 129.92, p<0.001, cf. Table S4). Self-attribution was lowest for ±30° deviations at 6.0±2.0%. The mean subjective threshold was at 14.7±1.1° corroborating our previous data in an independent participant pool. Motor awareness thresholds tended to be higher in the DT condition (16.7±1.6°; main effect of Task: (F(1, 13) = 4.1304, p = .063) and there was a significant interaction between factors Task and Deviation (F(4, 52) = 3.5567, p = .012). Post-hoc analysis revealed that this interaction was driven by a significant increase in erroneous self-attributions for angular deviations of ±10° and ±15° corresponding to the point of highest uncertainty (t-tests, p<0.001 and p<0.01 respectively, cf. Table S5).

Motor awareness during non-deviated trials was not significantly affected by the independent variables (Target Side (p>0.75), Target Position (p>0.53), all interactions p>0.11).

MA for deviated trials was sensitive to the position of the target. MA was more accurate when walking towards the inside than when walking towards the outside targets (main effect of Target Position: F(1, 13) = 16.185, p = .00145).

Unlike for the control trials, we observed a small but significant effect of deviation side on participants' MA. Participants had a higher error rate when deviations were to the left of the target, forcing participants to compensate towards the right (main effect of Deviation Side: F(1, 13) = 6.7930, p = .02174). Target Side did not have a significant effect (p>0.23).

Furthermore, there was an interaction between factors Target Side, Target Position, and Deviation Side (F(1, 13) = 32.632, p = .00007). Participants made more attribution errors when walking towards the outside targets, if the compensation was towards the inside, i.e. when walking towards the leftmost (rightmost) target with a deviation to the left (right). This relationship was flipped for the inside targets. Here participants reported higher attribution, if the deviation was towards the centre of the screen and they compensated outwards. This interaction resembles the one observed for MP and illustrates that participants made more MA errors, if MP was more accurate, minimizing the error in the visual feedback.

#### Response Time

Response Times were recorded but the emphasis was placed on response accuracy. Trials with RTs larger than 10 seconds and trials more than 3SD from the mean were excluded. RTs were not significantly affected by the main independent variables of Task (p>0.64) and Deviation (p>0.26). Average RT was 1476±142 ms (ST) and 1521±145 ms (DT). There was also no interaction between the two factors (p>0.16).

In control trials, RTs for the inside targets were significantly lower than for the outside targets (main effect of Target Position: F(1, 13) = 20.262, p = .0006) as they increased from 1276±113 ms (inside) to 1549±154 ms (outside). RTs did not depend on the Target Side (p>0.39, all interaction p>0.37).

Response Times in deviated trials were not affected by the Target Side (p>0.24) or the side of the deviation (p>0.70). RTs for deviated trials showed a similar main effect of Target Position (F(1, 13) = 6.6187, p = 0.02318) as the control trials. RTs were lower for the inside targets compared to the outside targets (RT-inside: 1484±142 ms, RT-outside: 1603±144 ms). Furthermore there was a significant interaction between factors Target Side and Deviation Side (F(1, 13) = 10.950, p = 0.00565). Participants responded faster when both target and deviation were on the same side and participants compensated by walking towards the midline. No other significant interactions were observed (all p>0.019).

#### Summary Motor Awareness

In summary, participants reliably recognised feedback in non-deviated control trials to be self-generated. This identification with the movement of the virtual body was not affected by cognitive loading or by the position and side of the target. Self-attribution decreased with increasing angular deviations and was lowest for deviations of 30°, confirming the participants' ability to correctly reject strongly deviated trials. Motor awareness thresholds (at the 50% level) increased under cognitive loading. MA for deviations around the threshold (10° and 15°) reflect the highest uncertainty and MA in these trials was significantly affected by cognitive loading. Participants thus judged significantly more deviated feedback to be non-deviated. MA in deviated trials was further susceptible to the position of the target as participants made less erroneous self-attributions when walking towards the inside targets than when walking towards the outside targets.

## Discussion

The purpose of our study was to investigate how cognitive loading affects goal-directed walking and motor awareness in healthy participants in a VR environment. Our results illustrate that the participants' walking accuracy and their walking trajectories were not affected by the secondary task even though taxing cognitive resources significantly decreased their walking velocity. In the DT condition, these changes were accompanied by impairments in motor awareness in trials with 10°–15° angular deviation, corresponding to the stimuli with the highest perceptual uncertainty. In the following we discuss our findings with respect to cognitive control of locomotion, common mechanisms underlying different forms of goal-directed behaviour and the use of visual movement feedback in neurorehabilitation.

### Sensorimotor and cognitive aspects of goal-directed walking

The trajectory deviations of ∼10 cm for goal-directed walking in a VR environment correspond to the range of deviation, i.e. 10–15 cm, previously reported for goal-directed walking in a natural environment [10], [11]. This suggests that our participants used similar stereotyped trajectories in order to reach the different target locations in control trials but also when they compensated for the range of angular deviations. Our data extend previous findings as they illustrate that adding a secondary task did not significantly affect the participants' trajectory deviations with respect to their own average trajectory (average deviation) in either case. The significant effect of cognitive loading on the maximum trajectory deviation is due to the fact that the calculation takes the timing of the average trajectory into account, which significantly changed with the walking velocity. This is supported by our findings on the trajectory error (ATE), which is calculated with respect to an ideal compensatory and time-independent trajectory. The ATE was not affected by cognitive loading. The stable results observed for the motor compensation and its accuracy, corroborate the above points suggesting that the mechanisms underlying goal-directed behaviour [12] are highly automated and require little cognition, at least during movement execution. This is inline with existing literature stating that locomotor trajectories may be predictively controlled [23], [24], i.e. planned in a feed-forward manner, and overlaid on an automated locomotor pattern [25]. The current paradigm highlights that this process was not affected by the cognitive load, even when participants had to make reactive changes [26] to their planned trajectories by incorporating on-going visual feedback.

Unlike for walking trajectories, cognitive loading had a strong impact on walking kinematics. Participants significantly slowed down while performing the secondary task, in-line with previous findings [15]. Further analysis revealed that this was not an effect of initial hesitation or freezing of gait, but that velocity was lower throughout the entire trial. Cognitive loading interfered with cortical mechanisms involved in maintaining the sequential locomotion pattern. We propose that these mechanisms are separate from the spatial aspects of the trajectory formation, which were not affected by taxing cognitive resources. The decrease in velocity could also reflect a task prioritisation, in which the participants favoured walking accuracy over maintaining walking velocity even though participants were instructed to walk at the same pace in both conditions. One limitation in the current paradigm was that the walking trajectories were limited to 1.8 m and came from a standing start. Future experiments should for one include longer walking trajectories to address if cognitive loading affects other spatiotemporal gait parameters cf. [12] and, for another, investigate other secondary tasks, such as a visuospatial processing task. Parameters such as head and trunk rotation deserve further examination as the decline of axial rotation presents a marker of early Parkinson's disease [27].

### Motor awareness and rehabilitation using virtual reality technologies

VR methods are becoming increasingly important tools for research, ranging from motor performance to neuroscience [28], as well as for therapy and rehabilitation. These methods can offer naturalistic scenarios while providing the therapist with high levels of adaptability and control [29], [30]. The use of real-time multimodal feedback may present an important opportunity as one can monitor and improve one's movements in real-time, but also from a neuroscience and neuro-rehabilitation perspective. Observing an action facilitates the brain's motor circuits involved in performing the same action [31] and this has been reported to depend on the familiarity with the observed action [32] and whether one attributes that action to oneself or another [33]. It is therefore important to understand under what conditions one recognizes one's own movements as self-generated during VR exposure and maintains a feeling of being in control of one's avatar (relating to the *sense of agency* [13], [34] and the concept of *presence* in a virtual environment [35]).

Our results are important as they illustrate the limits of self-attribution for deviated feedback and show that self-attribution for veridical feedback (0° deviation) and for strongly deviated feedback (30°) was not affected by cognitive load. However, motor awareness was more strongly impaired for selective deviated feedback-trials (around the threshold of ±10° to ±15°) when our participants were performing a cognitive task [36]. In case of perceptual uncertainty about the feedback, cognitive loading not only impaired motor control but also motor awareness for one's on-going movements.

### Motor Performance and Motor Awareness in Goal-directed Movements

As outlined in the introduction, striking similarities have been reported between the trajectories for saccadic eye-, arm-, and whole-body movements [8], [9]. Both control of such goal-directed actions, here MP, and conscious monitoring thereof [37], here MA, are understood to rely on a central monitoring framework [38]. This framework comprises of a comparator mechanism between internal representations and predictions about our movements, using the efference copy, along with the feedback we continuously receive about those movements [39]. Dating back to the physiological mechanisms of corollary discharge introduced by Sperry [40], von Holst and Mittelstaedt [41] and previously Helmholtz [42], these mechanisms form the basis for currently applied frameworks for sensorimotor control [43].

Our current results for goal-directed walking are comparable to studies performed for upper-limb movements [14], [37] in that participants automatically corrected for introduced angular deviations yet were not aware of these mismatches unless they were above ∼15°. This is important for two reasons. For one, these findings favour a general control strategy employed by the CNS to generate goal-directed behaviour, both predictive (control trials) and reactive (deviated trials), in an effector-independent manner. According to the comparator mechanism, the error resulting from the visually deviated feedback is automatically integrated and used to correct one's movement trajectory as evidenced by the motor compensation. For another, the results obtained for MA, suggest that motor awareness may similarly rely on an effector-independent and supramodal mechanism [34] as comparable paradigms have now been conducted for movements of fingers [44]–[47], hands [48]–[50] and arms [51]–[53] using both visual and auditory feedback. Our current findings along with [13], and recent findings on temporally delayed auditory [54] and visual feedback [36] during over ground and treadmill walking respectively have extended these paradigms to movements of the entire body.

The selective effects of cognitive loading on specific aspects of motor performance, i.e. walking velocity but not trajectories, as well as its strong modulation of motor awareness in threshold trials is further important as they point to distinct cortical and subcortical mechanisms involved in these tasks. With respect to motor awareness and action attribution, imaging studies have revealed a widespread neural network. There is a sensorimotor component including supplementary motor areas (pre-SMA and SMA), ventral pre-motor cortex (PMC) and the Cerebellum (CB) as well as a second component comprising of the posterior parietal cortex (PPC), temporo-parietal junction (TPJ), extrastriate body area, insula, anterior cingulate (ACC) and dorso-lateral pre-frontal cortex (PFC) [55]. In particular, the PFC, along with TPJ, SMA, PMC and ACC, has been linked to increased activation during the perception of spatiotemporal sensorimotor conflicts [56], [57], and error monitoring in general [58], and is most likely additionally burdened by the secondary task [59]. Our findings that motor awareness was selectively affected by cognitive loading in trials with 10° to 15° deviations indicate that the arithmetic task competed for these resources and interfered with the motor awareness task only in trials corresponding to the highest perceptual ambiguity.

Human locomotion is controlled by a hierarchical supraspinal locomotor network encompassing cortical regions including PFC, SMA and PMC, overlapping with the network described above, as well as subcortical regions such as the basal ganglia (BG), cerebellum and brainstem [28]. Importantly, these cortical regions that are affected by the dual task have been identified to control volitional aspects of locomotion such as gait initiation, termination and changes in direction or velocity during treadmill walking [60]–[63], and form part of the basal ganglia thalamo-cortical loop [64], [65]. The reduced walking velocities we report here are in-line with the dual tasking literature and indicate that cognitive loading interfered with the highly automated locomotor pattern, independent of the introduced angular deviations and the generation of the goal-directed walking trajectories.

### Conclusion

In conclusion, our data propose that goal-directed aspects of locomotion, the underlying kinematics and the conscious awareness thereof, all involve separable cortical (and subcortical) mechanisms. This is evidenced by the differential effects of cognitive loading: movement kinematics were uniformly affected by the secondary task, motor awareness only in trials with high perceptual uncertainty, whereas trajectory formation was not affected at all, at least in our participant pool. In the current study no singular gait parameter directly reflected the changes observed in motor awareness. More data are therefore needed to grasp how these different levels of sensorimotor control interact. One approach will be to extend the current paradigm to elderly subjects more strongly affected by dual tasking. The changes in walking trajectories, kinematics and movement awareness could potentially be used to separate frail and fit elderly participants and lead to a better understanding of sensorimotor control and awareness for locomotion. This will be central to developing complex intervention and rehabilitation strategies and could potentially shed light on cognitive markers of risk of falling in an elderly population.

## Supporting Information

Figure S1 **Experimental Procedure.**
(TIF)

Figure S2 **Dependent Gait Variables.**
(TIF)

Table S1 **Motor Compensation.** Posthoc Comparisons.
(DOCX)

Table S2 **Walking Times.** Posthoc Comparisons.
(DOCX)

Table S3 **Neck Yaw.** Posthoc Comparisons.
(DOCX)

Table S4 **Motor Awareness.** Posthoc Comparisons.
(DOCX)

Table S5 **Motor Awareness.** Interaction between Task and Deviation.
(DOCX)
