# Supplementary figures and images for: Cognitive Loading Affects Motor Awareness and Movement Kinematics but Not Locomotor Trajectories during Goal-Directed Walking in a Virtual Reality Environment
Source: PLoS One. 2014 Jan 21;9(1):e85560. doi: 10.1371/journal.pone.0085560 (PMC3897484; doi:10.1371/journal.pone.0085560)

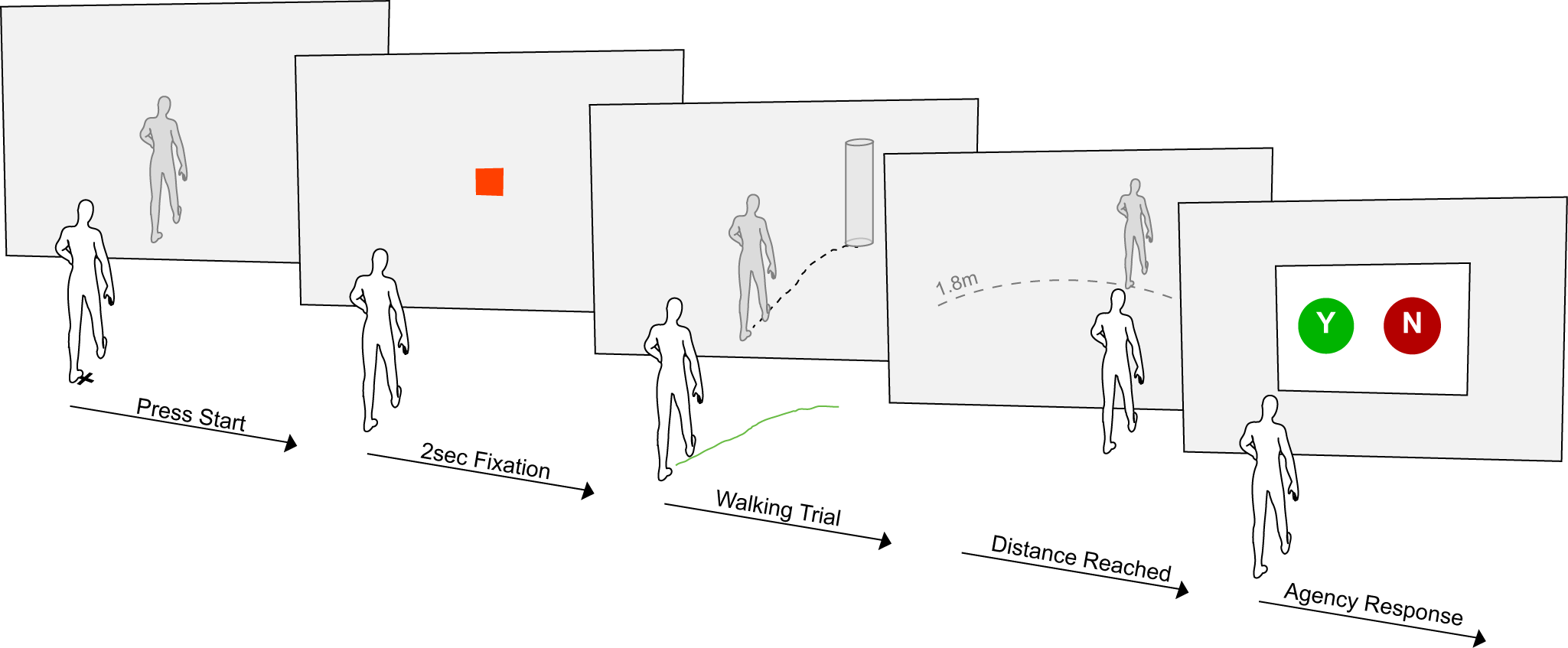

Supplement: Figure S1 — Experimental Procedure. (TIF) [file pone.0085560.s001.tif]

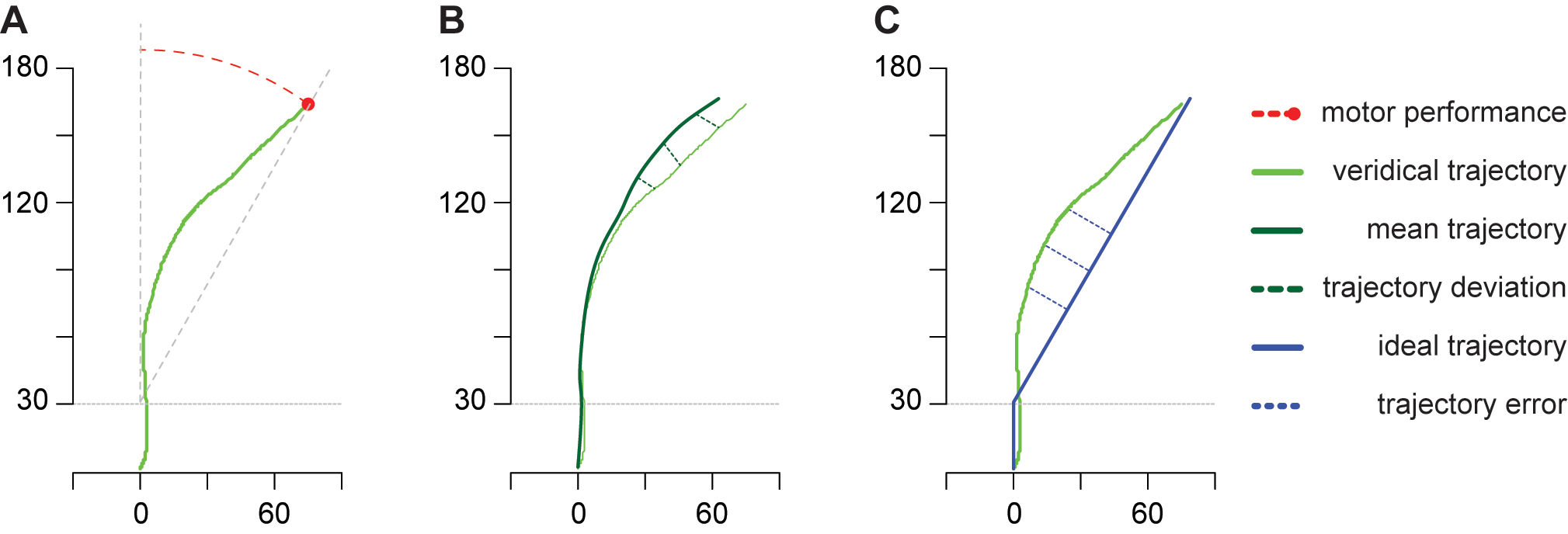

Supplement: Figure S2 — Dependent Gait Variables. (TIF) [file pone.0085560.s002.tif]
