# Supplementary material for: Cognitive Loading Affects Motor Awareness and Movement Kinematics but Not Locomotor Trajectories during Goal-Directed Walking in a Virtual Reality Environment
Source: PLoS One. 2014 Jan 21;9(1):e85560. doi: 10.1371/journal.pone.0085560 (PMC3897484; doi:10.1371/journal.pone.0085560)
Supplement: Table S2 — Walking Times. Posthoc Comparisons. (DOCX) [file pone.0085560.s004.docx]

|  | Fisher LSD | | | | | Bonferroni Corrected | | | | |
| --- | --- | --- | --- | --- | --- | --- | --- | --- | --- | --- |
| **Dev.** | 0º | 5º | 10º | 15º | 30º | 0º | 5º | 10º | 15º | 30º |
| 0º |  | **<0.001** | **<0.001** | **<0.001** | **<0.001** |  | **<0.001** | **<0.001** | **<0.001** | **<0.001** |
| 5º | **<0.001** |  | 0.941 | 0.588 | **0.012** | **<0.001** |  | 1.000 | 1.000 | 0.122 |
| 10º | **<0.001** | 0.940 |  | 0.640 | **0.015** | **<0.001** | 1.000 |  | 1.000 | 0.148 |
| 15º | **<0.001** | 0.588 | 0.640 |  | **0.045** | **<0.001** | 1.000 | 1.000 |  | 0.454 |
| 30º | **<0.001** | **0.012** | **0.015** | **0.045** |  | **<0.001** | 0.122 | 0.148 | 0.454 |  |

**Supplementary Table S2 : Walking Times** – Posthoc comparisons, significant differences are in bold face.
