# Supplementary material for: Cognitive Loading Affects Motor Awareness and Movement Kinematics but Not Locomotor Trajectories during Goal-Directed Walking in a Virtual Reality Environment
Source: PLoS One. 2014 Jan 21;9(1):e85560. doi: 10.1371/journal.pone.0085560 (PMC3897484; doi:10.1371/journal.pone.0085560)
Supplement: Table S3 — Neck Yaw. Posthoc Comparisons. (DOCX) [file pone.0085560.s005.docx]

| **Deviation** | 0º | 5º | 10º | 15º | 30º |
| --- | --- | --- | --- | --- | --- |
| 0º |  | 1.000 | 1.000 | 1.000 | **<0.001** |
| 5º | 1.000 |  | 1.000 | 1.000 | **0.0016** |
| 10º | 1.000 | 1.000 |  | 1.000 | **0.005** |
| 15º | 1.000 | 1.000 | 1.000 |  | **0.016** |
| 30º | **<0.001** | **0.0016** | **0.005** | **0.016** |  |

**Supplementary Table S3 : Neck Yaw** – Posthoc comparisons with applied Bonferroni Correction, significant differences in bold type.
