# Supplementary material for: Cognitive Loading Affects Motor Awareness and Movement Kinematics but Not Locomotor Trajectories during Goal-Directed Walking in a Virtual Reality Environment
Source: PLoS One. 2014 Jan 21;9(1):e85560. doi: 10.1371/journal.pone.0085560 (PMC3897484; doi:10.1371/journal.pone.0085560)
Supplement: Table S4 — Motor Awareness. Posthoc Comparisons. (DOCX) [file pone.0085560.s006.docx]

| **Deviation** | 0º | 5º | 10º | 15º | 30º |
| --- | --- | --- | --- | --- | --- |
| 0º |  | 1.000 | <0.001 | <0.001 | <0.001 |
| 5º | 1.000 |  | 0.0013 | <0.001 | <0.001 |
| 10º | <0.001 | **0.0013** |  | <0.001 | <0.001 |
| 15º | <0.001 | <0.001 | **<0.001** |  | <0.001 |
| 30º | <0.001 | <0.001 | <0.001 | **<0.001** |  |

**Supplementary Table S4 : Motor Awareness** – Posthoc comparisons with applied Bonferroni Correction. Planned comparisons highlighted in gray.
