# Supplementary material for: Cognitive Loading Affects Motor Awareness and Movement Kinematics but Not Locomotor Trajectories during Goal-Directed Walking in a Virtual Reality Environment
Source: PLoS One. 2014 Jan 21;9(1):e85560. doi: 10.1371/journal.pone.0085560 (PMC3897484; doi:10.1371/journal.pone.0085560)
Supplement: Table S5 — Motor Awareness. Interaction between Task and Deviation. (DOCX) [file pone.0085560.s007.docx]

|  |  | **Single Task** | | | | |
| --- | --- | --- | --- | --- | --- | --- |
|  | **Deviation** | 0º | 5º | 10º | 15º | 30º |
| **Dual Task** | 0º | 0.961 | 0.519 | <0.001 | <0.001 | <0.001 |
|  | 5º | 0.117 | 0.325 | <0.001 | <0.001 | <0.001 |
|  | 10º | <0.001 | <0.001 | **<0.001** | <0.001 | <0.001 |
|  | 15º | <0.001 | <0.001 | <0.001 | **0.009** | <0.001 |
|  | 30º | <0.001 | <0.001 | <0.001 | <0.001 | 0.342972 |

**Supplementary Table S5 : Motor Awareness : Interaction between Task and Deviation** – Posthoc comparisons, planned comparisons highlighted in gray. Trials around the MA thresholds, i.e. 10º and 15º, were significantly affected by the cognitive load.
